# Conductive Polymer Coatings Control Reaction Selectivity in All‐Iron Redox Flow Batteries

**DOI:** 10.1002/adma.202414596

**Published:** 2025-04-01

**Authors:** Emre B. Boz, Ameya Bondre, Ronald de Bruijne, Antoni Forner‐Cuenca

**Affiliations:** ^1^ Electrochemical Materials and Systems Department of Chemical Engineering and Chemistry Eindhoven University of Technology P.O. Box 513 Eindhoven 5600 MB The Netherlands; ^2^ Eindhoven Institute for Renewable Energy Systems Eindhoven University of Technology P.O. Box 513 Eindhoven 5600 MB The Netherlands

**Keywords:** all‐iron redox flow batteries, conductive polymers, hydrogen evolution, PEDOT, polypyrrole, porous electrodes, reaction selectivity

## Abstract

Aqueous all‐iron redox flow batteries are an attractive and economic technology for grid‐scale energy storage owing to their use of abundant and environmentally benign iron as the redox active material and water as solvent. However, the battery operation is challenged by the plating/stripping reactions of iron and the competing hydrogen evolution reaction at the negative electrode, which hinder performance and durability. Here, the reaction selectivity of the negative electrode is tailored by introducing conductive polymer coatings onto porous carbonaceous electrodes. Two conductive polymers, poly(3,4‐ethylenedioxythiophene) (PEDOT) and poly(pyrrole) (PPy) are conformally coated with the dopant poly(4‐styrenesulfonate) (PSS) and the resulting electrochemistry is studied on model electroanalytical platforms and redox flow batteries. Both polymers decrease the hydrogen evolution current on rotating disc electrodes, with PPy/PSS strongly inhibiting the reaction at high overpotentials. In full all‐iron redox flow cells, PPy/PSS coating extends cyclability and significantly reduces hydrogen evolution, while PEDOT/PSS coating improves the round‐trip efficiency, possibly acting as a redox shuttle for the iron stripping reaction. These findings motivate broader investigation and implementation of conductive polymers to engineer reaction selectivity for flow batteries and other electrochemical technologies.

## Graphical abstract

Aqueous redox flow batteries are limited by the competing hydrogen evolution reaction (HER) at their negative electrodes. In this work, conductive polymers are conformally coated on porous carbonaceous electrodes to improve the reaction selectivity of hybrid all‐iron redox flow batteries. Poly(pyrrole)/PSS coating inhibits HER in acidic media and increases the battery lifetime, while PEDOT/PSS electrode improves the roundtrip efficiency.

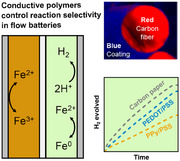

## 1 Introduction

**Figure 1:**
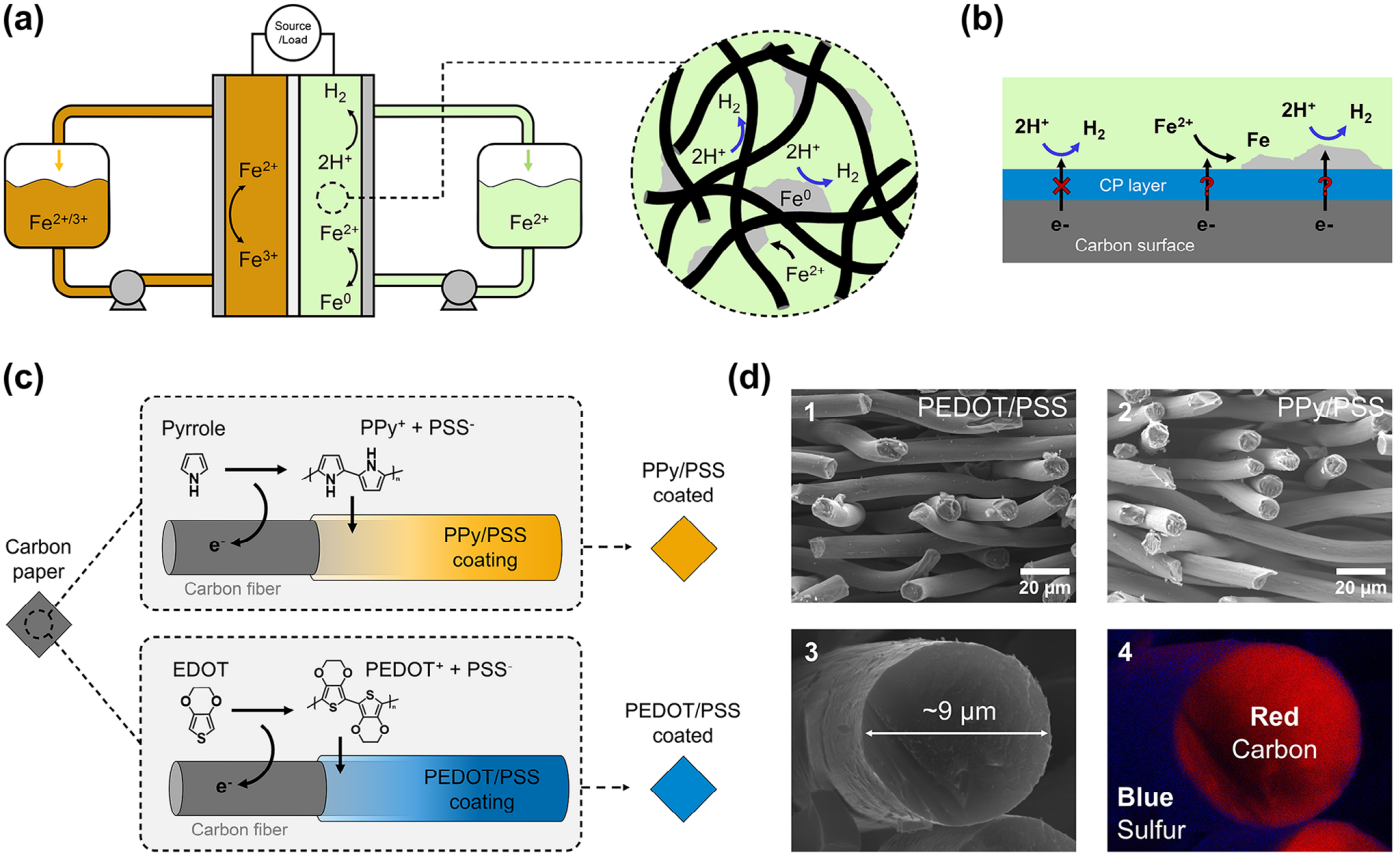
Conductive polymers to engineer reaction selectivity in all‐iron redox flow batteries. (a) Scheme of all‐iron hybrid flow batteries. In the zoomed‐in region, reactions at the negative electrode during charging are depicted. (b) The surface of a conductive polymer‐coated carbon electrode shows a possible route for reaction selectivity during the iron plating reaction. (c) Electropolymerization scheme employed in this study where oxidation of pyrrole or EDOT monomers in the presence of PSS^−^ polyanions result in PPy/PSS and PEDOT/PSS coatings on carbon paper electrodes, respectively. (d) Cross‐section SEM images of (1) PEDOT/PSS and (2) PPy/PSS coated carbon paper electrodes. (3) High‐resolution cross‐section SEM image of a carbon fiber within the PEDOT/PSS coated electrode and (4) EDX mapping of the same fiber showing the sulfur‐rich coating (blue) in contrast with the carbon‐rich fiber core (red).

Large‐scale energy storage technologies are key to provide operational stability in an energy grid with a large share of intermittent renewable sources such as wind and solar. Especially for developing countries and remote areas, increased penetration of renewable sources can threaten the grid reliability, and here affordable and sustainable storage technologies can make a positive impact.^[^ ^1^ ^]^ Redox flow batteries (RFBs) offer attractive properties for large‐scale energy storage such as long life‐cycles, modular design, and easily scalable storage capacity without scaling the cost of the entire battery system.^[^ ^2^, ^3^ ^]^ RFBs interconvert chemical energy stored in redox species into electrical energy in an electrochemical flow reactor during charge and discharge. RFBs can be classified based on the redox species employed, and the choice of electrolyte strongly influences the power density, safety, and the system cost.^[^ ^4^ ^]^ Consequently, the ideal flow battery chemistry for a sustainable and affordable system features high electrochemical performance with abundant raw materials that are environmentally safe and non‐toxic, among other characteristics.^[^ ^5^ ^]^ From this perspective, aqueous all‐iron redox flow batteries (AIRFBs) stand out as they combine the inherent safety of aqueous electrolytes with low‐cost and earth‐abundant iron as redox species. Furthermore, the use of the same species in both negative and positive electrolytes results in manageable species crossover similar to the commercial all vanadium‐based systems.^[^ ^6^ ^]^ The AIRFB (**Figure** **1**a) employs iron in its three oxidation states, *Fe* ^0^, *Fe* ^2 +^, and *Fe* ^3 +^ and features an open circuit voltage of ≈1.21 V at standard conditions (Equation 1). Because the negative side of the battery utilizes plating/stripping reactions of iron (Equation 2), the energy capacity is coupled to the electrode area/volume unless flowable electrodes are used,^[^ ^7^ ^]^ making this a hybrid‐type of flow battery. Similar to the zinc‐based hybrid flow batteries, the high density and two electron reaction of the iron metal results in elevated theoretical capacity (7558 mAh cm^−3^) of the negative side,^[^ ^8^ ^]^ but complicates battery operation due to the phase change reactions within the porous electrode.

(1) $$Cell\;reaction\;:\;3Fe^{2+}⇌Fe^{0}+2Fe^{3+}\;E^{0}=+1.21\;V$$

(2) $$Negative\;electrode\;:\;Fe^{2+}+2e^{-}⇌Fe^{0}\;E^{0}=-0.44\;V\;vs\;SHE$$

(3) $$Positive\;electrode\;:\;Fe^{2+}⇌Fe^{3+}+1e^{-}\;E^{0}=+0.77\;V\;vs\;SHE$$

The negative side of the AIRFBs is limiting the battery in terms of performance and lifetime due to the competitive hydrogen evolution reaction (HER) (Figure 1a). The standard potential of iron deposition is 440 mV below HER (*E* ^0^ =  0 *V* *vs* *Standard Hydrogen Electrode*, *SHE*), and iron demonstrates a higher exchange current density than graphite,^[^ ^9^, ^10^ ^]^ causing faradaic losses and capacity imbalance during battery operation. Electrolyte pH can be increased to limit proton availability and increase the overpotential for HER; however, the solution should be acidic to prevent precipitation of the ferrous ions.^[^ ^11^ ^]^ During operation, the negative electrode continuously consumes protons (or generates hydroxides) and shifts the equilibrium to form more ferrous hydroxide, which is electrochemically inactive and sparingly soluble, resulting in capacity fade.^[^ ^12^ ^]^ Eventually, the cell components (e.g., porous electrodes, membrane, flow field channels, tubes) start clogging and this cascade of events causes battery death.^[^ ^12^ ^]^ The parasitic HER also affects the positive electrolyte, as the oxidation of Fe^2+^ to Fe^3+^ on the positive side sustains the charge spent on HER on the negative side. This leads to capacity imbalance, causing Fe^3+^ to accumulate in the positive electrolyte. To improve the battery lifetime, the negative tank can be rebalanced through acidification or, alternatively, the excess Fe^3+^ of the positive side can be spontaneously reduced to Fe^2+^ with the hydrogen side‐product in a recombination reactor.^[^ ^12^, ^13^ ^]^ Both strategies are viable but neither addresses the faradaic efficiency decrease due to HER. Ideally, the reaction selectivity of the negative electrode should be improved to ensure long operation times with minimal intervention.

Electrolyte engineering strategies can improve the reaction selectivity of AIRFBs, however, the limited pH window and the abundance of proton donors in the electrolyte creates a challenging medium to completely mitigate HER. Various electrolyte additives have been explored to alter the solvation structure of iron ions or to act as buffering agents.^[^ ^14^, ^15^, ^16^, ^17^, ^18^, ^19^, ^20^ ^]^ Coordination of ferrous iron to citrate can improve the stability of the electrolyte with a negative impact on the solubility and OCV of the battery.^[^ ^16^ ^]^ Similarly, ligands such as glycine can prevent hydrolysis of ferric ions at the positive side but can interfere with the plating/stripping reactions at the negative side.^[^ ^14^ ^]^ Solvents such as DMSO can replace water molecules in the solvation shell of iron and decrease the water activity, resulting in high‐capacity retention; however organic solvents can negatively impact electrolyte viscosity and conductivity, and may decrease the solubility of the redox species.^[^ ^18^ ^]^ Alternatively, reaction selectivity can be tackled at the electrode‐electrolyte interface, without significantly changing the bulk properties of the electrolyte. Certain metals with completely filled d‐orbitals (e.g., Zn, Cd, Hg, In, Sn, Pb, and Bi) do not favor hydrogen chemisorption and have shown low exchange current densities for HER.^[^ ^21^, ^22^ ^]^ When added to the electrolyte in low amounts (in their salt form), metals that are more noble than iron can create a surface layer on the electrode, and the HER current can be decreased during iron plating in acidic media as demonstrated by *in‐situ* electrodeposition of Cd and In.^[^ ^15^, ^23^ ^]^ Although HER‐inhibiting metals can extend the water stability window, they tend to be rare or toxic and may negatively impact the recyclability of the electrolyte. To fulfill the sustainability, affordability, and safety criteria of AIRFBs, electrode modification strategies should focus on abundant and non‐toxic elements.

Organic molecules offer a more sustainable and tailorable route for electrode functionalization. Functional molecules that can form molecular films on the substrate via adsorption, polymerization, and electrografting have been utilized to improve reaction selectivity for aqueous reduction reactions.^[^ ^24^, ^25^, ^26^ ^]^ The modified interfaces for HER inhibition are hypothesized to block water from reaching the surface by increasing the surface hydrophobicity or to limit proton/electron availability by forming insulating layers.^[^ ^27^, ^28^ ^]^ Self‐assembled monolayers of n‐alkylthiols and vapor‐deposited PTFE have been successfully employed to improve surface hydrophobicity for iron anodes and nitrogen reduction electrocatalysts.^[^ ^29^, ^30^ ^]^ Conductive polymers (CPs) are another class of organic coatings that have semiconducting properties and can suppress HER on metals in acid media.^[^ ^31^, ^32^ ^]^ In fact CPs have been used as corrosion protection coatings in acidic solutions, where the coating is applied to create a *metal‐CP‐solution* interface and the corrosion protection can be attributed to a physical barrier effect or passivation of the metal.^[^ ^33^, ^34^ ^]^ Given their ability to modulate interfacial properties, we posit that CPs could also play a role in controlling reaction selectivity in hybrid redox flow batteries. However, the situation is different for an AIRFB as a *CP‐metal‐solution* interface will be formed during battery operation. Then the challenge of CPs for AIRFBs is preventing the plated iron layer from nullifying the coating functionality, as iron acts as a catalyst for HER. Semiconductors (and conductive polymers) also influence the electronic structure of the metal by modulating its work function, thereby changing the reactivity of the surface.^[^ ^35^, ^36^ ^]^ Although such interfacial effects would be localized at the metal‐semiconductor junction and not extend into the bulk of the metal layer, reduced HER rates have been observed on bulk metals electrodeposited on deeply reduced CPs.^[^ ^37^ ^]^ Thus the semi‐insulating nature of CPs at cathodic potentials can still impact the electron availability for the particles deposited on it, though it is not yet clear if such electron deficiency will impact the plating kinetics of iron (Figure 1b). Nevertheless, we hypothesize that CP coatings can be particularly useful in limiting the HER after a deep discharge compared to an uncoated substrate commonly used for AIRFBs such as steel meshes or carbon fiber mats. Many RFB chemistries suffer from side‐reactions, such as HER for all‐vanadium and zinc‐based batteries, and electrode‐enhanced ferricyanide reduction for aqueous organics, resulting in capacity decay and material degradation.^[^ ^38^, ^39^ ^]^ We propose that conductive polymer coatings can simplify system operation and extend the battery lifetime by targeting the reaction selectivity of porous electrodes.

In this work, we explore, for the first time, the application of conductive polymer coatings on porous electrodes to suppress hydrogen evolution in redox flow batteries. We studied two conductive polymers, polypyrrole (PPy) and poly(3,4‐ethylenedioxythiophene) (PEDOT) doped with a large anion poly(4‐styrenesulfonate) (PSS^−^) (Figure 1c). PEDOT and PPy are well‐studied conductive polymers with good chemical stability and high conductivity, while the smooth film‐forming ability of PSS^−^ in the conductive polymer network is beneficial for applications with forced liquid convection. We have previously demonstrated that PEDOT/PSS can be conformally coated on porous electrodes (Freudenberg H23) with a tunable thickness via electropolymerization from aqueous solutions and here we extended the same technique to deposit PPy/PSS (Figure 1d).^[^ ^40^ ^]^ First, we investigate the electrochemical properties of the polymers on glassy carbon electrodes in acidic media with rotating disc electrode experiments. Second, we study the charge and discharge polarization performance of coated and uncoated carbon paper electrodes in full‐cell configuration. Finally, we perform cyclic charge‐discharge experiments with simultaneous measurement of the hydrogen gas evolved in the negative electrode with in‐line gas chromatography (GC). We show that the developed electrode interfaces can tune the reaction selectivity for iron plating reaction in AIRFBs without a significant impact on the battery performance, or improve the performance without impacting the reaction selectivity. We envision that the results from this work can assist other aqueous electrochemical reduction technologies necessary for a sustainable and electrified energy economy such as nitrogen reduction, CO_2_ reduction, and metal‐air batteries.

## 2 Experimental Section

### 2.1 Chemicals

3,4‐ethylenedioxythiophene (EDOT, 97%, Fluorochem), pyrrole (98%, Sigma Aldrich), poly(sodium 4‐styrenesulfonate) (PSSNa, 500–700 kDa, 20.4 wt.%, Tosoh Organic Chemical Co.), sodium chloride (NaCl, Sanal P+, >99%), hydrochloric acid (HCl, Boom, 37–38%), iron(II) chloride tetrahydrate (FeCl_2_·4H_2_O, ≥99.0%, Thermo Scientific) and iron(III) chloride hexahydrate (FeCl_3_·6H_2_O, ≥99.0%, Sigma Aldrich) were used without further purification. All instances of water refer to ultrapure water (18.2 MΩ, Millipore).

### 2.2 Electropolymerization Substrates and Setup

Porous carbon electrodes (Freudenberg H23 carbon paper, cut in 2 cm by 2 cm square shape, 210 µm nominal thickness, 80% porosity, QuinTech) or glassy carbon electrodes (GCEs) in rotating disk form (RDE, Pine Instruments, 0.196 cm^2^) were used as the electropolymerization substrates. To ensure proper wettability in aqueous monomer solutions, the carbon paper electrodes were heat‐treated in a muffle furnace (Nabertherm) under air atmosphere with a heating ramp of 20 °C min^−1^ until 450 °C, kept at 450 °C for 12 h and naturally cooled down to room temperature. All instances of carbon paper refer to heat‐treated carbon paper. The electrical connection between the porous substrates and the potentiostat leads was made with a gold foil‐covered crocodile to prevent corrosion of the electrical connections. GCEs were polished to mirror finish with 0.05 µm alumina slurry on a polishing pad before coating. Carbon paper and GCEs were used as the working electrodes, carbon paper as the counter electrode and silver‐silver chloride electrode (BASi, Ag/AgCl in 3 m KCl) as the reference electrodes.

### 2.3 Electropolymerization of PEDOT and PPy

Aqueous solutions were prepared with EDOT (0.01 m) or pyrrole (0.1 m) as the monomers and PSSNa (0.1 m) as the supporting salt, and the resulting polymers are named as PEDOT/PSS or PPy/PSS, respectively. The molarity of PSSNa was calculated based on the molarity of the repeating unit (sodium 4‐vinylbenzenesulfonate). The high viscosity of the monomer solution may cause air bubbles to get trapped in the carbon paper electrode, thus the electrodes were immersed in the aqueous monomer solution and were briefly sonicated in a bath sonicator (≈10 s) right before electropolymerization. The reactions were carried out in ≈75 mL of the monomer solutions at room temperature.

#### 2.3.1 Carbon Paper Substrates

Based on our previous work, for electropolymerization of PEDOT/PSS and PPy/PSS on carbon paper, 3.2 mA (0.8 mA cm^−2^ considering the geometric area) is passed from the cell for 25 min in constant current mode, and the resulting potential responses can be found in Figure S1a (Supporting Information).^[^ ^40^ ^]^ The coated electrodes were extensively washed with water before the experiments.

#### 2.3.2 Glassy Carbon Substrates

The porous nature of the carbon paper makes it difficult to match the current densities between substrates, thus we opted for constant potential electropolymerization for coatings on GCEs. The potential is selected to match the plateau potential of the reactions on carbon paper, which are ≈1 V for EDOT and ≈0.55 V for pyrrole. The potential is lower in the case of pyrrole owing to its higher concentration and lower standard reduction potential than EDOT.^[^ ^41^ ^]^ The potential was held at their respective values until 0.05 C has passed for both polymers (Figure S1b, Supporting Information). Additionally, to study the effect of polymer thickness on HER, PPy/PSS was coated at various charges (0.002, 0.01, 0.05, and 0.1 C) by varying the polymerization time (Figure S1c, Supporting Information). For the constant potential mode, automatic iR compensation was applied where the voltage was corrected for the solution resistance extracted at 100 kHz with 20 mV sinus amplitude and 85% of the resistance was compensated. The RDE was not rotated during electropolymerization. The coated electrodes were extensively washed with water before the experiments.

### 2.4 Microscopy

Cross‐section micrographs at 1KX magnification (see Figure 1d, panel 1 and 2) were obtained with a JEOL JSM IT‐100 scanning electron microscope (SEM) operating at 10 kV accelerating voltage and 10 mm of working distance. To prepare the cross‐section samples, coated electrodes were cut from the middle with rapid impact of a sharp razor. Elemental compositions were determined with an EDX detector housed within the SEM chamber at the same conditions. Cross‐section image zoomed in on a single fiber coated with PEDOT‐PSS (see Figure 1d, panel 3) was obtained with a FEI Sirion UHR field emission SEM at 17KX magnification, 5 mm working distance and 20 kV accelerating voltage. EDX mapping on this fiber (see Figure 1d, panel 4) was performed with an EDAX Octane Pro detector housed within the SEM chamber at the same conditions.

### 2.5 RDE Experiments

GCE in a rotating disc format was used as the working electrode to study the influence of coatings on the hydrogen evolution reaction. The RDE was rotated at 900 rpm and linear sweep voltammetry (LSV) was performed at 2 mV s^−1^ scan rate in the cathodic direction. 2 m NaCl solution (acidified to pH≈2 with 1 m HCl) was used as the electrolyte, carbon paper as the counter electrode and reversible hydrogen electrode (HydroFlex, Gaskatel GmbH) as the reference electrode. To ensure standard potential of hydrogen is not influenced by concentration effects, solutions were purged with hydrogen for 20 min before the experiments and the headspace of the electrochemical cells were purged with hydrogen during the experiments. Three linear sweeps were performed successively, and the third sweep is reported to eliminate the influence of surface oxides and polymer reduction on the HER current. For the HER experiments, the surface of the GCE was either bare, coated with a polymer, plated with iron, or coated with a polymer and then plated with iron.

#### 2.5.1 Iron Plating on GCEs

To plate iron on the coated or bare GCEs, 0.5 m FeCl_2_·4H_2_O in 2 m NaCl solution (pH≈2) was used as the electrolyte and carbon paper was used as both the counter and reference electrodes in a 2‐electrode setup. 5 mA cm^−2^ (cathodic) was applied on the working electrode until 0.53 C has passed, resulting in a theoretical plating thickness of 1 µm (assuming density of α‐iron as 7.86 g cm^−3^ and a homogenous coating with no side reactions). The resulting potential responses can be found in Figure S2a (Supporting Information). The RDE was not rotated during plating reactions. The plated electrodes were extensively washed with water before the experiments.

### 2.6 Redox Flow Battery Setup

All‐iron hybrid flow batteries were conducted with a prototype‐size redox flow cell.^[^ ^42^ ^]^ The flow diffusers of the cell were machined from polypropylene (McMaster‐Carr) and the graphite flow fields, also acting as current collectors, were milled from 3.18 mm thick impregnated graphite (G347B graphite, MWI, Inc.). Interdigitated flow fields (IDFF) were used to facilitate the electrolyte flow into the porous electrode and feature seven dead‐ended channels, of which four are inlet and three are outlet channels and each channel is 1.6 cm in length, 1 mm in width and 0.5 mm in depth. The positive electrode compartment of the cell housed a bare carbon paper electrode, and the negative compartment housed a bare or polymer‐coated carbon paper electrode. The electrodes were cut to 1.7 cm by 1.5 cm (2.55 cm^2^ geometric area) and were enclosed within two incompressible polytetrafluorethylene gaskets (nominal thickness 55 and 110 µm, total thickness 165 µm, ERIKS). The positive and negative compartments were separated with an anion exchange membrane (FAP‐450, Fumasep). The cells were compressed by applying 2 N m torque, and final electrode compression was set to ≈21% by stacking two gaskets on each side. The cells were recompressed after 20 min to account for the mechanical relaxation of the cell body.

Electrolytes with redox‐active iron species were prepared with an acidified NaCl solution (pH≈2.5) to prevent hydroxide formation during mixing. For all RFB experiments, the electrolyte was pumped into the cell using a peristaltic pump (Cole‐Parmer) and rubber tubes (Masterflex Versilon, LS/14, ID = 1.6 mm).

For polarization tests, 0.5 m FeCl_2_·4H_2_O in 2 m NaCl solution (pH≈2.3) was used as the negolyte and the posolyte. Each compartment was connected to separate electrolyte tanks that held 5 mL of electrolyte pumped at 40 mL min^−1^. The electrolyte was purged with humidified Ar before the experiments, but during the experiments the tanks were not purged or sealed.

For cycling tests, 0.5 m FeCl_2_·4H_2_O in 2 m NaCl solution (pH≈2–2.3) was used as the negolyte and 0.25 m FeCl_2_·4H_2_O + 0.25 m FeCl_3_·6H_2_O in 2 m NaCl solution (50% pre‐charged, pH≈1.37) as the posolyte. Each compartment was connected to separate electrolyte tanks (Pyrex, GL‐45 thread) that held 50 mL of electrolyte pumped at 20 mL min^−1^. The headspace of the posolyte tank was continuously purged with humidified Ar during experiments.

### 2.7 Hydrogen Quantification

#### 2.7.1 In‐Line GC Setup

To quantify the H_2_ produced during cycling, the negolyte tank was sealed with a liquid distributor lid, housing 4 screw joints (BOLA); two for the electrolyte inlet/outlet, a gas inlet for the dry N_2_ flowing at 10 mL min^−1^, and a gas outlet. The screw connection was sealed with PTFE tape and checked for leaks after assembly. The tubes entering the connectors were rigid perfluoroalkoxy alkane (PFA, BOLA, OD = 3.2 mm, ID = 1.6 mm) and the electrolyte carrying PFA tubes were coupled to flexible rubber tubes for proper function of the peristaltic pump and had the same inner diameter (see Figure S3a, Supporting Information for a photograph of the setup). The N_2_ flow into the negolyte tank was controlled by a mass flow controller (Brooks, 200 sscm full range). The gas outlet is connected to a gas chromatograph (GAS, Compact GC 4.0) with a Molsieve 5A and RT‐Q‐BOND columns and a TCD detector with N_2_ as the carrier gas for H_2_, and He as the carrier gas for the N_2_ detection channel. The calibration curve for H_2_ can be found in Figure S4 (Supporting Information). The calibration was verified before each experiment with the negolyte tank under N_2_ flow and connected to the cell but without electrochemical input.

#### 2.7.2 Inverted Cylinder (Water Displacement) Setup

To measure the volume of hydrogen evolved during the long cycling tests, the negolyte tank was sealed with a gas‐tight lid, housing three compression‐type tube connectors (BOLA, 3x GL‐14 thread), two for the electrolyte inlet/outlet and one for the gas outlet. The gas outlet tube was placed in an inverted graduated cylinder (100 mL) filled with water that was stationed in a larger water reservoir (400 mL) and the evolved gas was collected in the headspace of the cylinder over the course of the experiments (see Figure S3b, Supporting Information for a photograph of the setup). The gas volume was recorded periodically and is reported without any corrections.

### 2.8 Battery Testing Protocols

#### 2.8.1 Discharge Polarization

The cells were initially charged at 20 mA cm^−2^ until the cutoff voltage of 1.8 V was reached, and discharged at stepwise increasing current densities for 2 min. The cells were charged with the same protocol between every discharge step.

#### 2.8.2 Charge Polarization

The cells were initially charged and discharged at 20 mA cm^−2^ until the cutoff voltages of 1.8 and 0.2 V were reached, respectively, and then charged at stepwise increasing current densities for 2 min. The cells were discharged with the same protocol between every charge step. The median voltage of the cells at the corresponding current density was taken as the equilibrium voltage to plot the polarization curves.

#### 2.8.3 Cycling

The cells were charged at 25 mA cm^−2^ until 15 mAh has passed (≈14.1 min) or until the upper cutoff is reached (1.8 V) and discharged at the same current density until lower cutoff is reached (0.2 V). 50 cycles were run for tests with the in‐line GC setup, while for longer cycling tests the cells were run until the capacity decayed substantially and reached a plateau.

## 3 Results and Discussion

### 3.1 Hydrogen Evolution Reaction on Coated Electrodes

**Figure 2:**
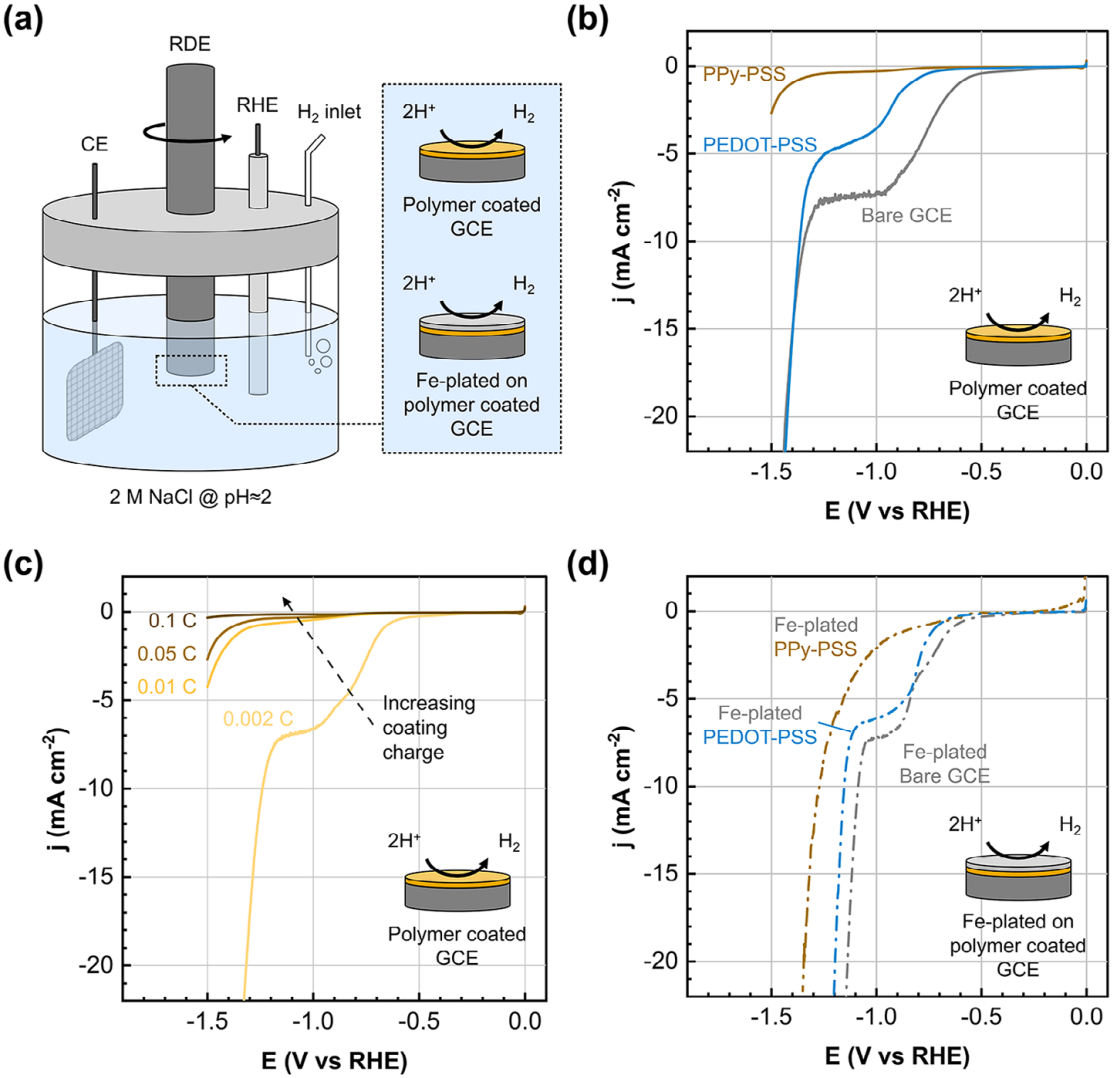
PPy/PSS coating hinders HER in the absence and presence of iron. (a) Schematic of the RDE setup where linear sweep voltammetry is conducted on coated and/or plated GCEs. Voltammograms of (b) bare, PEDOT/PSS, and PPy/PSS coated electrodes (polymerization charge = 0.05 C), (c) PPy/PSS coated electrodes at polymerization charges of 0.002, 0.01, 0.05, and 0.1 C, and (d) bare and coated electrodes (polymerization charge = 0.05 C) that are electroplated with iron (plating charge = 0.53 C). All RDE experiments are conducted on GCEs having 0.196 cm^−2^ surface area at 900 rpm versus RHE in 2 m NaCl at pH≈2 with H_2_ purging/blanketing before/during the experiments, respectively.

We first investigate the influence of the conductive polymer interfaces toward hydrogen evolution using model electroanalytical platforms. Although porous electrodes are used in practical reactor architectures in RFBs, their complex geometry results in ill‐defined hydrodynamic conditions for analytical experiments, thus we opt for glassy carbon as a substrate material and use the rotating disc electrode format to induce well‐defined mass transfer conditions.^[^ ^43^ ^]^ HER is reported to be sensitive to the “electron availability” at the surface and a deeply reduced CP layer could hinder electron transfer and reduce the reaction rate.^[^ ^27^ ^]^ To test whether the conductive polymer coatings are effective in inhibiting HER, we perform linear sweep voltammetry in supporting electrolyte solutions (at pH≈2) on polymer‐coated GCEs (**Figure** **2**a). Considering that iron metal demonstrates a high exchange current for HER,^[^ ^10^ ^]^ and will be present on the electrode surface during battery operation, we also performed LSVs on polymer‐coated electrodes that are electroplated with iron (Figure 2a).

First, we compare the influence of the PEDOT/PSS and PPy/PSS coatings on HER rates in acidic media (Figure 2b). The electrodes are coated by passing the same amount of charge (0.05 C) (see Figure S1b, Supporting Information for the current‐time plots). When the electrodes are polarized to potentials below 0 V (vs RHE) at pH≈2, we expect to see a limiting current response for proton reduction, followed by alkalinization of the near‐surface region and eventually water reduction to become the dominant HER mechanism.^[^ ^44^, ^45^, ^46^, ^47^ ^]^ The bare GCE and the PEDOT/PSS coated GCE clearly follows this trend (Figure 2b), where the limiting region (from −0.9 V to −1.3 V vs RHE) is followed by a steep water reduction current. It seems that the PEDOT/PSS coated electrode is effective in decreasing the proton reduction rate as seen with its higher onset potential and lower limiting current but has no influence on the water reduction step. The PPy/PSS coating on the other hand results in order‐of‐magnitude lower currents than the bare and PEDOT/PSS coated electrodes, demonstrating a strongly inhibiting effect for HER throughout the potential window. Furthermore, the features in the voltammogram of PPy/PSS coated GCEs (see the zoomed‐in plot in Figure S5a, Supporting Information) resemble that of the bare and PEDOT/PSS coated GCEs, hinting that the presence of a CP layer does not significantly change the HER reaction steps.

At this point, it is not clear if the difference between the two CP coatings is due to an intrinsic material property (such as electronic conductivity) or due to their morphological properties (such as porosity), as it is uncertain where the electron transfer reactions occur on a coated electrode. Previous reports on PPy‐coated metal electrodes indicate that electroactive cations such as ferrocenium and [Os(bpy)_3_]^2+^ react at a sub‐micrometer thick polymer layer near the solution.^[^ ^48^ ^]^ For a reduced CP coating with low electrical conductivity, this would necessitate charge transfer within the polymer layer, which would be the rate‐limiting step (i.e., redox polymer model). Another possibility is the diffusion of protons and water through the coating and charge transfer occurring at the glassy carbon interface.^[^ ^31^, ^49^ ^]^ If the polymer layers are thin enough (<1 µm), redox reactions at this interface could sustain the current, which would require proton or water transport through a sodiated PSS‐network followed by the removal of the evolved hydrogen gas through the polymer layer (membrane model). For even thinner layers (<10 nm), electron tunneling effects could play a role in the charge transfer reactions.^[^ ^50^ ^]^ It has been reported that for electropolymerization of poly(pyrrole), 24 mC cm^−2^ passed charge corresponds to ≈0.1 µm thick coating on Pt electrodes.^[^ ^49^ ^]^ Considering the polymerization charge in our RDE experiments (254 mC cm^−2^) and the bulky PSS counterion in the structure, we expect the coating thickness on GCEs to be >1 µm. Thus, the tunneling mechanism does not play a role in the overall reaction rate.

To understand the influence of polymer thickness on the HER currents, we varied the polymerization charge using PPy/PSS as the baseline. By changing the polymerization charge we can control the thickness of the coating (Figure S1c, Supporting Information), and we verify this by comparing the reduction waves of the polymers in their first LSV cycle (Figure S5b, Supporting Information), as a thicker coating would require a larger cathodic charge to be completely reduced. At a polymerization charge of 0.002 C (10 mC cm^−2^), the voltammogram is almost identical to the bare GCE (Figure 2c) with a slightly lower limiting current. Interestingly, for such a thin polymer coating the overpotential for water reduction is lower than the bare electrode by ≈110 mV, which could be due to improved hydrophilicity of the electrode surface owing to the PSS within the polymer blend. For all other cases, the HER current decreases with increasing polymerization charge for both reaction steps. Although a clear limiting current region cannot be observed in all cases, the reciprocal current at −1.1 V (vs RHE) scales linearly with the polymerization charge (Figure S5c, Supporting Information), and this trend was previously ascribed to a diffusion limitation of protons within the polymer film.^[^ ^31^ ^]^ However, a charge transfer limitation within the polymer layer would manifest itself in a similar way,^[^ ^51^ ^]^ and it is likely that both processes contribute to the limiting current. Deconvoluting the rate‐controlling step of HER on the polymer films is outside the scope of the current work, but we envision that independently varying the coating thickness, rotation speed, and analyte concentration in controlled experiments can be instrumental to reach a more mechanistic understanding.

Finally, we investigated whether a plated iron layer renders the coating properties obsolete by electroplating iron on the bare and coated substrates. The plating continued until 0.53 C has passed, which should deposit a 1µm thick Fe layer (Figure S2a, Supporting Information). The catalytic effect of iron for HER is apparent as lower overpotentials are observed on all samples, especially for the water reduction step (Figure 2d). The trends observed on Fe‐free samples are partly retained here with PPy/PSS showing the best performance. If the charge transfer is limited within the polymer, its reaction‐inhibiting effect could extend to the plated iron layer on top of it, since the electron transfer within iron would be fast. Nucleation analysis of the plating voltammograms (Figure S2c–e, Supporting Information) indicate that the HER rate during plating is different between the samples. This would change the amount of iron on the surface for coated and uncoated surfaces and because of this point we refrain from a detailed analysis of Figure 2d and instead opt for determining the HER rate during battery cycling in the next sections.

It is also possible that the plated layer is not homogenously distributed on the bare or coated glassy carbon surfaces, meaning that iron‐free surfaces may be exposed to the electrolyte. This could also be the case for the porous electrodes during battery operation and the coatings may be useful in inhibiting HER on exposed surfaces after a deep discharge. To understand the plating/stripping dynamics, we recorded images of the bare GCE (in plate form) during iron plating and stripping reactions under a microscope (Figure S6, Supporting Information). We observe that plating is uneven due to bubble attachment on the surface and that iron does not strip completely during discharge. In AIRFBs, the iron plating‐stripping reaction will be dominant under forced convection and will determine the overpotentials. Consequently, to understand the influence of polymers on overpotentials, we first perform polarization experiments in a realistic redox flow battery setting.

### 3.2 Polarization Experiments

The performance of an AIRFB strongly depends on the reversibility of the plating/stripping reaction, which prompts us to investigate the polarization of the redox flow batteries in full‐cell configuration. The full‐cell configuration consists of a carbon paper positive electrode and an uncoated or polymer‐coated carbon paper as the negative electrode. Unlike previously reported work for AIRFBs utilizing felt electrodes,^[^ ^12^, ^13^, ^16^, ^18^, ^23^, ^52^, ^53^ ^]^ we opted for carbon paper electrodes for both compartments. The use of carbon paper can lower ohmic resistance, reduce the overall stack volume, and benefit from a previously optimized electropolymerization protocol on carbon paper electrodes.^[^ ^40^ ^]^ The polymers are coated on carbon paper using a polymerization charge of 4.8 C (1.2 C cm^−2^_geo_) and details of the coating procedure can be found in the Experimental Section. We use uncoated carbon paper on the positive side since the Fe^2+/3+^ redox couple has facile and reversible kinetics on oxidized carbon electrodes.^[^ ^54^ ^]^ The compartments are separated with an anion‐exchange membrane and the posolyte and negolyte tanks each contain 5 mL of 0.5 m of FeCl_2_ (in 2 m NaCl at pH≈2.3) circulating at 40 mL min^−1^ through the cell.

**Figure 3:**
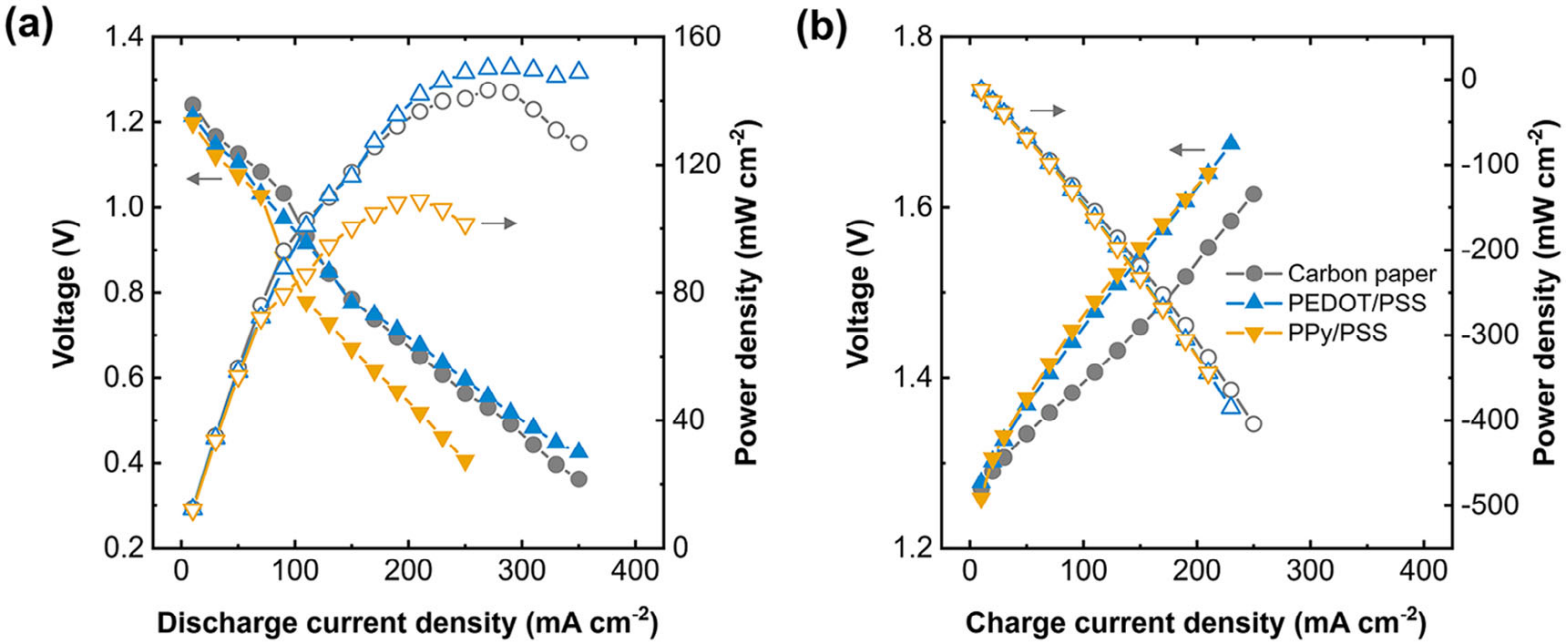
PEDOT/PSS coating improves the polarization performance. (a) Discharge and (b) charge polarization plots of the uncoated, PEDOT/PSS coated, and PPy/PSS coated carbon paper electrodes with the median voltages and the resulting power densities. Negative power density implies charging. The lines are to guide the eye.

For the discharge polarization tests, we fully charged the cells at 20 mA cm^−2^ (1.8 V cutoff) and then discharged at increasing current densities (0.2 V cutoff). We did not observe any solid particles in the negative tank, suggesting that the plated iron is electrically connected to the electrodes, which is desired for battery stability and lifetime.^[^ ^17^ ^]^ PPy/PSS coated carbon paper exhibits higher overpotentials, whereas PEDOT/PSS coated carbon paper outperforms the uncoated substrate especially at high current densities (**Figure** **3**a), resulting in ≈150 mW cm^−2^ peak power density at 300 mA cm^−2^ which is one of the highest reported values in the literature for hybrid AIRFBs.^[^ ^18^ ^]^ At high discharge current densities, the polymers may act as redox shuttles by performing their own oxidation reaction, which could facilitate the oxidation of the plated iron. The lack of a reference electrode means that the potential at the negative side cannot be probed directly, and the broad range of PEDOT oxidation further complicates this analysis.^[^ ^55^ ^]^ If we assume that the contribution of the negative side on the cell overpotentials is dominant, which is plausible considering the higher exchange current density of the Fe^2+/3+^ couple,^[^ ^43^, ^56^ ^]^ then the potential of the positive electrode would be close to the solution potential of the Fe^2+/3+^ redox couple (0.77 V vs SHE). This analysis suggests that the polymers can be electrochemically oxidized once the potential of the negative side reaches >0 V (<0.8 V for the cell voltage as an upper estimate). Since the oxidized form of PEDOT has higher electrical conductivity than its reduced state,^[^ ^55^ ^]^ the cell resistance would decrease during discharge, resulting in a higher power density. Consequently, the lower power density of the PPy/PSS during discharge can be ascribed to its lower conductivity in its oxidized state (reported with perchlorate as the dopant anion).^[^ ^57^ ^]^

To assess the battery performance during charging, we fully charged the cells at increasing current densities (1.8 V cutoff), and then fully discharged at 20 mA cm^−2^ (0.2 V cutoff) before the next measurement. We also fully charged and discharged the cells prior to the charge polarization experiments to condition the electrode surface. We observe that PPy/PSS demonstrates higher charging potentials across the entire current range while PEDOT/PSS is almost identical when compared to the uncoated carbon paper (Figure 3b). The polarization experiments suggest that the PEDOT/PSS coating can improve the voltage efficiency (VE) and energy efficiency (EE) of the battery during cycling, however the bottleneck in AIRFBs is the coulombic efficiency (CE) and battery lifetime as hydrogen evolution rapidly degrades the electrolyte. Consequently, a penalty on EE and VE can be afforded if the CE of the battery improves substantially, and in the next section we perform cycling experiments to compare the coated and bare substrates in a lab‐scale AIRFB setup.

### 3.3 Battery Cycling with In‐Line Gas Chromatography to Quantify H_2_ Production

**Figure 4:**
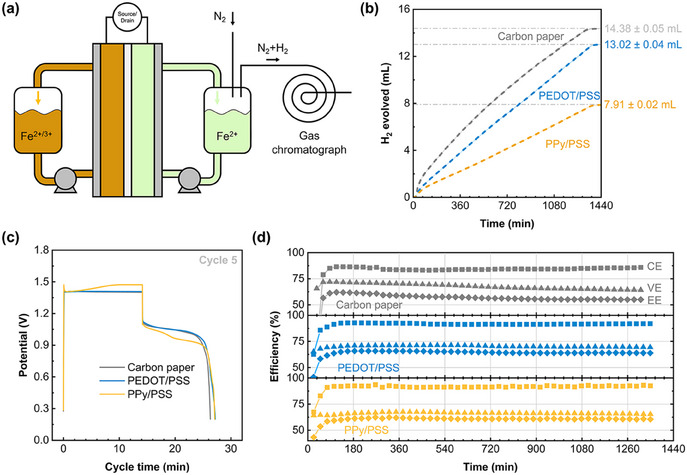
PPy/PSS inhibits HER with a voltage penalty. (a) Schematic description of the full cell setup with an in‐line GC for hydrogen amount quantification. (b) Evolved H_2_ volume plotted against time for uncoated, PEDOT/PSS, and PPy/PSS coated carbon paper electrodes. The total gas volume for each cell is denoted next to the plot. Errors are propagated from the error of the flow meter (shaded areas). (c) Potential‐cycle time curves for all cells at cycle 5. PPy/PSS has higher charge and discharge overpotentials than the other electrodes. (d) Efficiencies of all electrodes against time. The cells are cycled at 25 mA cm^−2^ with 15 mAh charge limit. The median efficiency values can be found in Table 1.

During AIRFB operation, the hydrogen gas accumulates in the headspace of the negolyte tank and diffuses out if the tank is not sealed. By measuring the gas flow, it is possible to calculate the share of the current density spent on HER. Considering this, we employ a gas‐tight tank for the negolyte (see Figure S3a, Supporting Information) having a gas inlet for nitrogen at a fixed flow rate (10 mL min^−1^) and a gas outlet carrying the evolved hydrogen and nitrogen mixture for further analysis to gas chromatography (**Figure** **4**a).

Before comparing the electrodes, we need to describe the operating conditions of the AIRFB setup used in this work. The negolyte is composed of 50 mL of 0.5 m FeCl_2_ in 2 m NaCl at pH≈2, while the posolyte is at 50% SoC (0.25 m FeCl_2_ + 0.25 m FeCl_3_) in 2 m NaCl at pH≈1.37. The cells are limited by their posolyte capacity (335 mAh posolyte vs 1340 mAh negolyte) and are cycled via constant current charge/discharge at 25 mA cm^−2^ with 15 mAh charge limit (with 1.8 V safety cutoff) and with 0.2 V discharge cutoff. We limited the charging cycles to 15 mAh to prevent clogging the reactor with solid iron, as 15 mAh corresponds to 30% reactor filling (after compression) for the carbon paper electrodes in this study. The charge imbalance between the compartments ensures that the negolyte side has abundant Fe^2+^ and hydrogen evolution is not favored due to mass transfer limitation of iron ions. For the in‐line GC setup, we tested the batteries for 50 cycles (≈23 h) to reach a steady state performance and H_2_ production rate. Longer cycling of the cells was not possible with the GC due to instrument availability, but we attempted to collect the evolved H_2_ with longer cycling of the cells (up to 72 h) in the next section.

The hydrogen production rate of the cells (Figure S7, Supporting Information) is calculated by measuring the H_2_ concentration in the carrier gas, which can be correlated to the HER rate of the cells. For all experiments, the hydrogen production rate is highest for the first cycle and quickly reaches a stable value. The share of the current density that is spent on HER also stabilizes ≈2% for the uncoated carbon paper and the PEDOT/PSS coating, and 1.16% for the PPy/PSS coating. Integration of the hydrogen production rate results in the evolved hydrogen volume as a function of time (Figure 4b). By the end of the 50 cycles, the cell with uncoated electrode generates 14.38 ± 0.05 mL of H_2_, PEDOT/PSS generates 13.02 ± 0.04 mL, and PPy/PSS evolves the lowest amount of H_2_ at 7.91 ± 0.02 mL. The trends in gas evolution follow the results of RDE experiments where PEDOT/PSS is not as efficient in preventing HER as PPy/PSS.

The impact of HER on battery operation is two‐fold. First, protons are consumed on the negative electrode, increasing the negolyte pH and causing hydroxide formation, which can cause membrane fouling and clogging of the tubes.^[^ ^6^ ^]^ Second, the charge spent on HER causes Fe^3+^ build‐up in the posolyte, meaning that less Fe^2+^ will be available for the consecutive charge cycle.^[^ ^6^ ^]^ During cycling experiments, we observed that the color of the posolyte changes from yellow/orange to dark orange/brown, indicating increasing concentration of Fe^3+^ chloride complexes.^[^ ^58^ ^]^ Simultaneously the negolyte solution turns from pale green to dark green accompanied by significant turbidity and precipitation in the tubes, suggesting that insoluble hydroxide species are forming due to increasing pH (see Figure S3c, Supporting Information for a photograph of the electrolyte tanks after longer cycling). Thus, it is clear that pH control in hybrid AIRFBs is a critical step for long‐term operation, and PPy/PSS coating emerges as a promising material for HER inhibition.

Aside from the battery lifetime, the energy and voltage efficiency are critical metrics as they relate to the overpotentials during charge and discharge cycles. Voltage response of the cells within one charge/discharge cycle can be found in Figure 4c. As expected from the polarization curves of the electrodes in Figure 3, PPy/PSS exhibits higher charge and discharge overpotentials compared to the uncoated and PEDOT/PSS coated electrodes. The performance of PEDOT/PSS coated and uncoated carbon paper electrode is similar in terms of overpotentials in cycle 5, however as seen in Figure 4d, the uncoated electrode undergoes a degradation in voltage efficiency while the coated electrodes are stable for 50 cycles. In fact, PEDOT/PSS coated electrodes feature higher voltage and energy efficiency than both uncoated and PPy/PSS coated electrodes, and a comparable coulombic efficiency with the latter (see **Table** **1** for the efficiency values) albeit with higher H_2_ production. Interestingly, the CE values do not directly correlate to the share of the current that is spent on HER (1–2%), implying that the current efficiency is dominated by processes other than the parasitic reactions. Microscopy during plating/stripping (Figure S6, Supporting Information) revealed that some iron remains on the surface and cannot be stripped at the same current density. We find that the porous electrodes are attracted by magnets after cycling tests, implying residual iron in the structure. Together with the pH‐induced precipitation in the negolyte tank, the residual iron may block the liquid pathways and result in battery failure. Thus the ideal electrode material must inhibit HER to protect the negolyte pH and facilitate iron stripping to keep the electrode pores open over the batteries lifetime. In the next section, we extend the battery cycling to investigate this cell failure phenomenon.

**Table 1:** Efficiencies of the cells with uncoated, PEDOT/PSS and PPy/PSS coated carbon paper electrodes. CE, VE, and EE are averaged over 50 cycles.

Sample	CE^a)^ [%]	VE^a)^ [%]	EE^a)^ [%]
Carbon paper	82.99 ± 10.48	67.73 ± 2.54	56.24 ± 7.42
PEDOT/PSS	91.05 ± 4.23	70.23 ± 1.03	63.97 ± 3.56
PPy/PSS	91.17 ± 3.70	66.24 ± 0.80	60.40 ± 2.75

^a)^ The errors are one standard deviation from the mean. The errors are amplified by the first cycles where all efficiencies are lower as HER is dominant.

### 3.4 Longer Cycling of the Batteries

**Figure 5:**
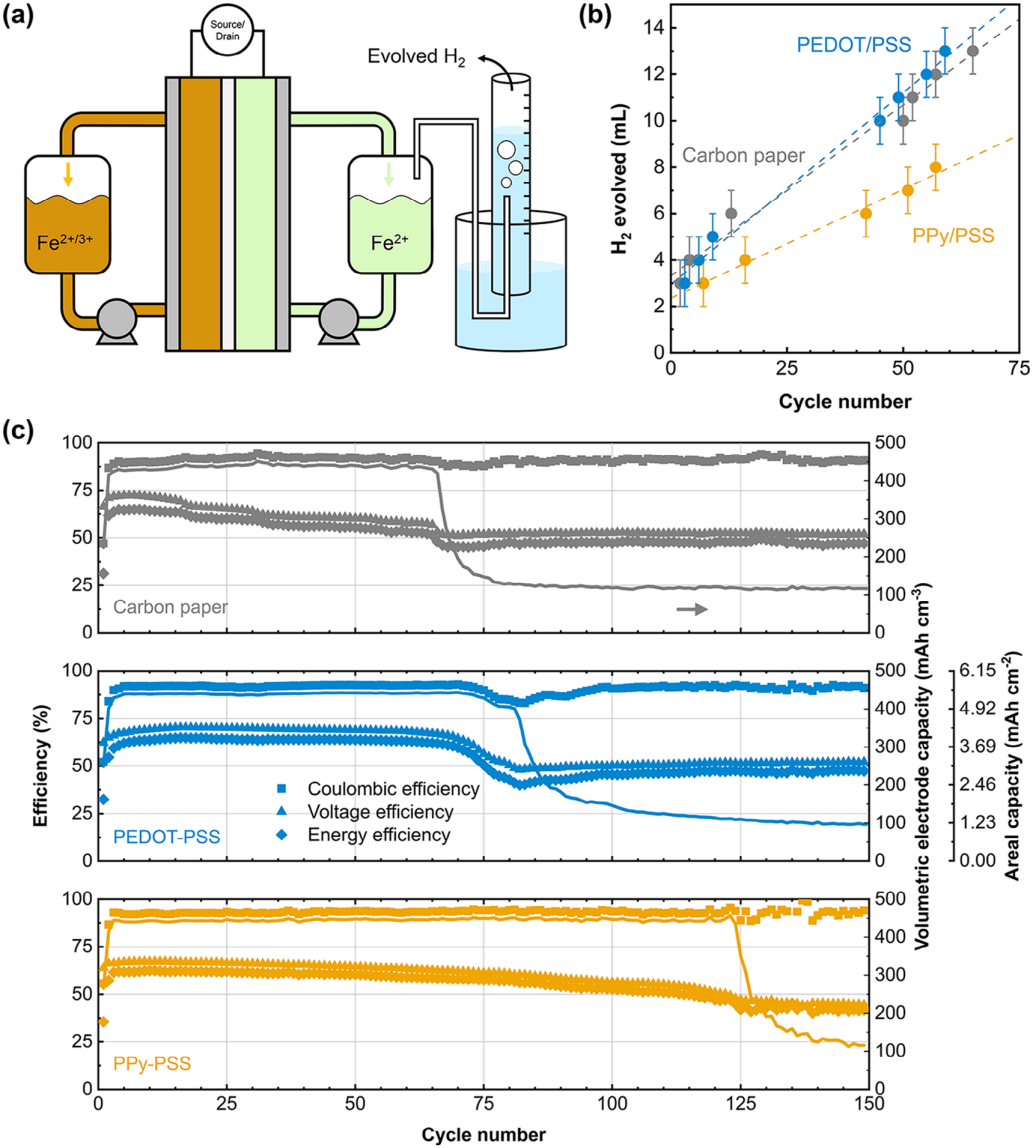
PPy/PSS coating improves battery lifetime. (a) Schematic description of the full cell setup with simultaneous gas collection from the negolyte headspace. (b) Evolved H_2_ volume plotted against the cycle number for uncoated, PEDOT/PSS, and PPy/PSS coated carbon paper electrodes. The dashed lines are linear fits of the scatter plots. Error bars are the reading error of the graduated cylinder. (c) Efficiencies and electrode free‐volume normalized (*volumetric electrode capacity*) and geometric area normalized (*areal capacity*) discharge capacities of all electrodes against cycle number. The cells are cycled at 25 mA cm^−2^ with 15 mAh charge limit. See Table S1 (Supporting Information) for averaged efficiencies.

Next, we cycled the cells until failure while capturing the evolved hydrogen. Unfortunately, excessive water injection at long cycling times into the hydrophilic GC column caused quantification problems, while the evaporation of the electrolyte (due to dry N_2_ flow) would be detrimental to the performance. To accommodate for this, we opted for collecting the evolved gas in a water‐filled inverted cylinder setup (**Figure** **5**a). This setup is less quantitative than the in‐line GC setup as the gas volume is recorded manually, and the system is more susceptible to leaks due to the slight negative pressure of the water column. Nevertheless, we observed similar trends with the long‐term cycling of the cells in terms of evolved hydrogen volume (Figure 5b).

To probe the cyclability of the cells, we use the decline of discharge capacity as an indicator. Due to the voltage cutoff during charging, the accessed capacity gradually decreases when the total cell potential increases to more than 1.8 V, and at ≈25% of the original capacity we assume that the cell has failed. It is common to use the areal charge/discharge capacities of hybrid flow batteries as a comparison metric as it relates to physical changes in the battery such as dendrite formation.^[^ ^59^ ^]^ However, this metric does not take electrode thickness into account, favoring the use of thicker mats such as felt and cloth electrodes. To normalize for the available volume for deposition reactions, we report *volumetric electrode capacity* as a complimentary metric, which is the discharge capacity per void space of the compressed electrode (Figure 5c). Then, for a common felt electrode used in RFBs (AvCarb G100) at 18% compression, 100 mAh cm^−2^ corresponds to an electrode volumetric capacity of 415 mAh cm^−3^ and a reactor filling (total solid phase in the compartment) of ≈11%. For the carbon paper electrodes used in this study, matching this areal capacity is not possible as 90% of the reactor would be filled at 80 mAh cm^−2^, causing significant pressure drop and transport limitations. Thus, we limited the charging cycles to 15 mAh (lasting ≈14 min), corresponding to an areal capacity of 5.88 mAh cm^−2^, volumetric electrode capacity of 478 mAh cm^−3^, and a reactor filling of 30%. Matching the volumetric capacities of the electrodes rather than their areal capacities allows a fair comparison between electrodes of different thicknesses. Finally, regardless of the electrode used, 100% reactor filling would be equal to the theoretical volumetric capacity of α‐iron (7558 mAh cm^−3^).

Assuming that 2% of the current is spent on HER (as determined with the in‐line GC setup for uncoated carbon paper), ≈0.6 mAh would be lost every charge/discharge cycle and the posolyte (335 mAh capacity) would be depleted from Fe^2+^ ions in ≈543 cycles. In our experiments, the capacity decay starts much earlier as the voltage cutoff for charging (1.8 V) is reached at 66 cycles for uncoated electrode, 82 cycles for PEDOT/PSS, and 125 cycles for PPy/PSS coated electrodes (Figure 5c; Figure S8, Supporting Information). From this estimation, we can conclude that the main failure mechanism of the cells is not HER‐induced posolyte depletion. Near the end of a cell's lifetime, we observe heavy pressure drop in the negative electrode compartment. It is likely that the residual iron and the precipitated hydroxides limit the mass transfer in the porous electrode and are responsible for the cell failure. The micrographs and elemental maps of the negative electrode after 50 cycles (Figure S9, Supporting Information) show that regions under the channels of the flow field contain abundant iron residues. While incomplete stripping of iron is one culprit for the solid mass in the electrode, iron hydroxide that forms in the electrode, or being pumped from the negolyte tank into the electrode, can also block the pore network. The increased cyclability of the battery with PPy/PSS coating suggests that the precipitation‐induced failure can be delayed by inhibiting HER, but more work is needed to elucidate the exact failure mechanism of the cells.

We hypothesize that PEDOT can act as a redox shuttle during the oxidation reactions and facilitate the iron stripping reaction (discharge) of the battery, but is still limited by its high HER rate. PPy/PSS on the other hand demonstrates superior cyclability than PEDOT/PSS (66 cycles vs 125 cycles to cell failure) while its round‐trip efficiency is lower in both cycling tests. Thus, we envision that PEDOT/PSS coatings could improve the battery efficiency for an AIRFB system with auxiliary units that can manage electrolyte degradation, such as recombination cells.^[^ ^12^, ^60^ ^]^ Meanwhile, standalone AIRFBs with very high CE (>99%) would not require electrolyte management systems, which can reduce the capital costs.^[^ ^6^ ^]^ While our results with PPy/PSS are promising, achieving higher CE and stronger HER inhibition (and thus, longer lifetime) would necessitate further optimization of the coating chemistry, morphology, and thickness.

Our work demonstrates the possibility to tune the reaction selectivity, lifetime, and performance of AIRFBs with conductive polymer coatings. Although it is not clear which charge‐transfer mechanism is dominant for PPy/PSS and PEDOT/PSS, future experiments could investigate the reaction selectivity as a function of coating thickness and counterion type to provide a deeper understanding of the mechanism. In this regard, experiments with liquid‐phase and outer‐sphere redox couples could provide valuable information, as the liquid‐phase redox reactions do not change the electronic structure of the surface (unlike iron plating reaction). Furthermore, techniques such as scanning electrochemical microscopy could elucidate the influence of coating thickness and conformality on HER currents and would be instrumental to study the plating dynamics on polymer surfaces. Finally, more research is needed to boost the charge reversibility of coated electrodes to >99% to enable long‐term operation. One solution could be using the coatings in tandem with HER‐inhibiting metal nanoparticles^[^ ^23^ ^]^ in ligand‐based electrolytes that can coordinate to iron ions^[^ ^15^ ^]^ to strike a balance in battery performance and lifetime.

## 4 Conclusion

Suppressing the hydrogen evolution side reaction is a key challenge for many electrochemical technologies that rely on cathodic reactions in aqueous media, all‐iron redox flow batteries being one prominent example. In this work, we coated porous carbonaceous electrodes with two conductive polymer blends, PEDOT/PSS and PPy/PSS, to improve the reaction selectivity of iron plating/stripping against hydrogen evolution reactions in acidic media. On flat model electrodes, PPy/PSS effectively suppresses hydrogen evolution while PEDOT/PSS only suppresses the proton reduction step. The polarization experiments with coated porous electrodes in full cells reveal that PPy/PSS suffers from increased charge and discharge overpotentials, while PEDOT/PSS electrode shows a remarkable power density of ≈150 mW cm^−2^ at ≈300 mA cm^−2^. To deconvolute HER from iron plating/stripping, we measure the hydrogen volume generated on the negative side during battery charge/discharge cycling with in‐line gas chromatography. PPy/PSS coated electrodes evolve the least amount of H_2_ and show improved cyclability in long‐term cycling experiments compared to uncoated and PEDOT/PSS coated electrodes, while PEDOT/PSS coating improves the voltage and energy efficiency of the battery. The improved reaction selectivity of PPy/PSS can be particularly promising for electrochemical technologies where the products can be removed from the surface such as CO_2_ and nitrogen electroreduction.

## Conflict of Interest

The authors declare no conflict of interest.

## Author Contributions

E.B.B. contributed to the conceptualization, methodology, formal analysis, investigation, data curation, writing‐original draft, writing‐review and editing, and visualization. A.B. and R.B. contributed to the investigation, data curation, and writing‐review and editing. Finally, A.F.‐C. contributed to the conceptualization, methodology, funding, resources, writing‐original draft, writing‐review and editing, project administration, and supervision.

## Supporting information



Supporting Information

## Data Availability

The data that support the findings of this study are available from the corresponding author upon reasonable request.
