# Supplementary material for: Conductive Polymer Coatings Control Reaction Selectivity in All‐Iron Redox Flow Batteries
Source: Adv Mater. 2025 Apr 1;37(26):2414596. doi: 10.1002/adma.202414596 (PMC12232242; doi:10.1002/adma.202414596)
Supplement: Supplementary file 1 — Supporting Information [file ADMA-37-2414596-s001.pdf]

ADVANCED MATERIALS

Supporting Information

for *Adv. Mater.*, DOI 10.1002/adma.202414596

Conductive Polymer Coatings Control Reaction Selectivity in All-Iron Redox Flow Batteries

*Emre B. Boz, Ameya Bondre, Ronald de Bruijne and Antoni Forner-Cuenca**

Supporting Information

Conductive Polymer Coatings Control Reaction Selectivity in All-Iron Redox Flow Batteries

*Emre B. Boz[1,2], Ameya Bondre[1], Ronald de Bruijne[1], Antoni Forner-Cuenca[1,2,]**

[1]Electrochemical Materials and Systems, Department of Chemical Engineering and Chemistry, Eindhoven University of Technology, P.O. Box 513, 5600 MB Eindhoven, The Netherlands

[2]Eindhoven Institute for Renewable Energy Systems, Eindhoven University of Technology, P.O. Box 513, 5600 MB Eindhoven, The Netherlands

**Corresponding author: a.forner.cuenca@tue.nl, +31 (0) 6 202 02 775*

Table of Contents

Section S1: Electropolymerization profiles on porous and flat substrates 3
Section S2: Voltage profiles and nucleation plots of Fe-plating on bare or coated GCEs 4
Section S3: Gas evolution measurement setups and electrolyte tanks after cycling 5
Section S4: Calibration curve of the GC for H_2 6
Section S5: HER experiments on PPy/PSS coated GCEs 7
Section S6: Plating/stripping dynamics under optical microscope 8
Section S7: Hydrogen production rate of the cells 9
Section S8: Long term cycling experiments 10
Section S9: Cross section SEM micrographs of an electrode after cycling 11

Section S1: Electropolymerization profiles on porous and flat substrates

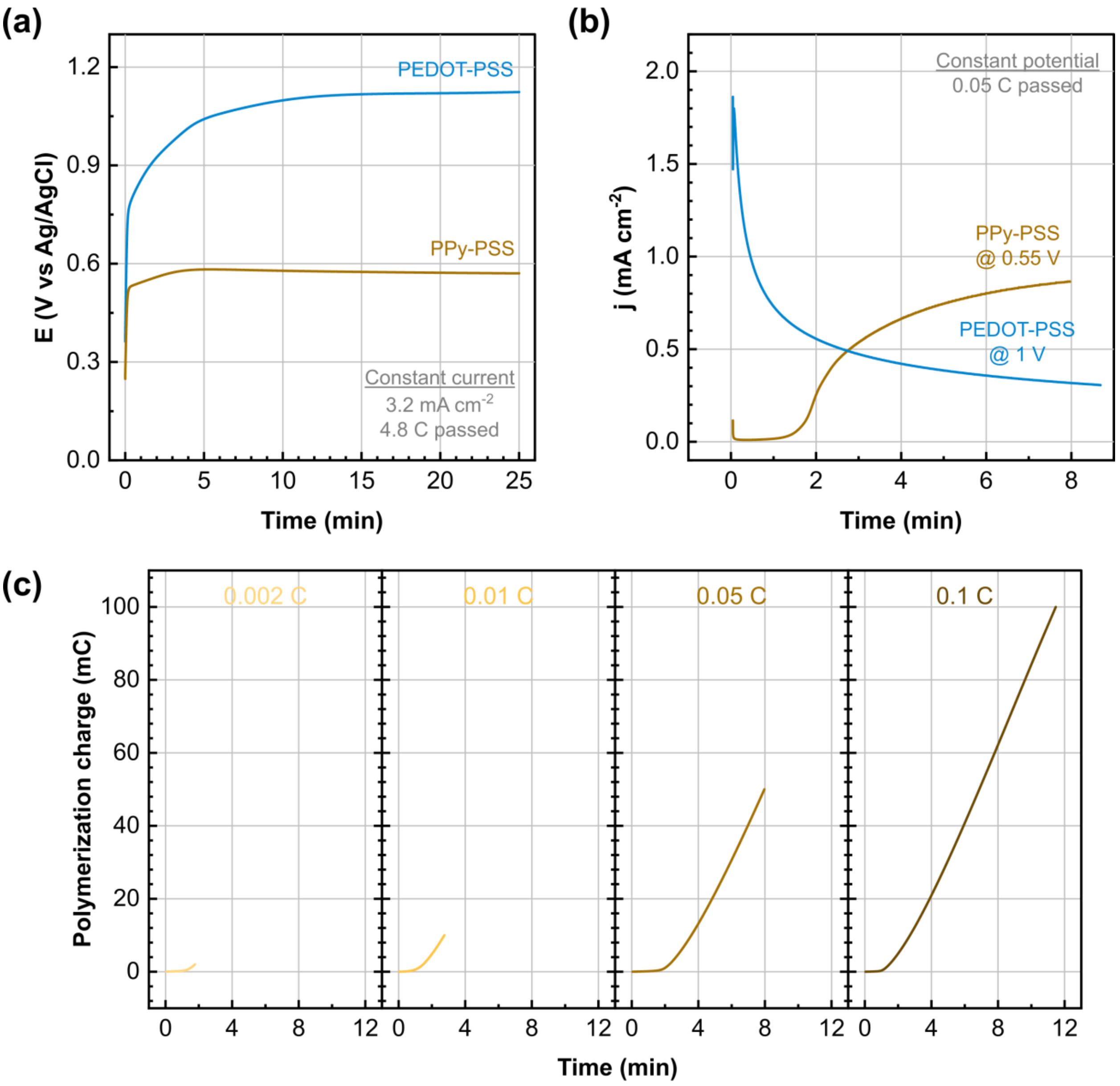

Figure S1. (a) Constant current (0.8 mA cm^{-2}) electropolymerization of PEDOT/PSS and PPy/PSS on carbon paper electrodes. **(b)** Constant potential electropolymerization of PEDOT/PSS and PPy/PSS on GCEs. The potential is 1 V for PEDOT/PSS and 0.55 V for PPy/PSS (*vs.* Ag/AgCl). The polymerization charge is 0.05 C. **(c)** Constant potential electropolymerization of PPy/PSS (0.55 V *vs.* Ag/AgCl) on GCEs at various polymerization charges.

Section S2: Voltage profiles and nucleation plots of Fe-plating on bare or coated GCEs

Based on previous reports on iron plating reaction on carbon electrodes, we expect progressive nucleation before t_{min} is reached, and instantaneous nucleation after t_{min}.[1] This holds true for all samples, although with deviations from the theoretical case. **Before t_{min}**, progressive nucleation can be observed on Bare GCE (**Figure S2c**), however the response is slightly delayed on polymer coated electrodes (**Figure S2d, S2e**). This has been ascribed to an induction time for nucleation in a study where silver nanoparticles were deposited on PPy electrodes.[2] However this induction time could also be due to the polymer reduction. Since polymers are oxidized as-coated, a small amount of charge would be used to reduce the polymer before iron plating reaction can take place. **After t_{min}**, the response of all samples are closer to the instantaneous nucleation model, but the responses strongly deviate at high t/t_{min}. Low faradaic efficiencies for iron plating could explain the observed deviation as the potentials are lower than expected by the S-H model.[1] The normalized voltage of Bare and PEDOT/PSS coated GCEs are close to each other and higher than PPy/PSS, in line with the observed H_2 evolution rates on full cells both via GC and inverted cylinder setup.

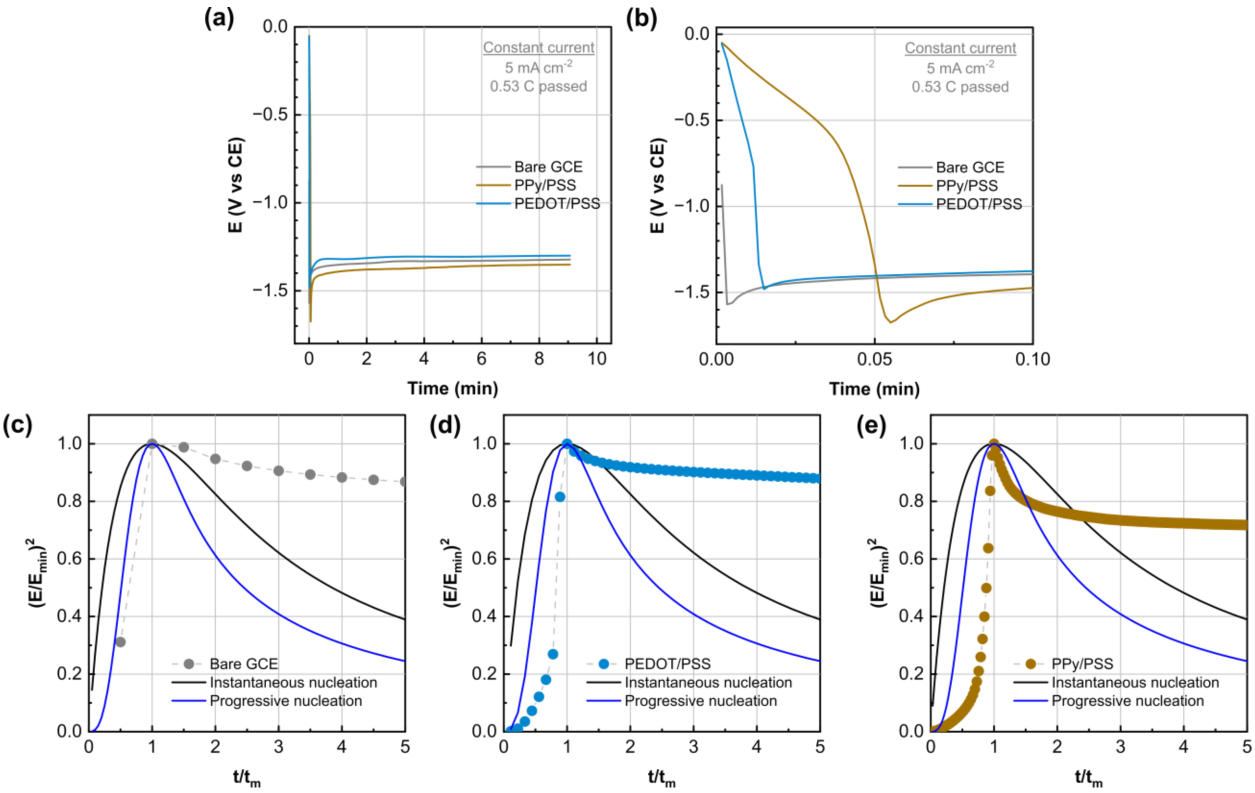

Figure S2. (a) Constant current (5 mA cm^{-2}) iron plating on bare or coated GCEs in a two-electrode setup where the counter electrode is carbon paper. The plating charge is 0.53 C. **(b)** The zoomed in plot of **(a)** to show the nucleation onset. Normalized voltage-normalized time plots of **(c)** Bare, **(d)** PEDOT/PSS and **(e)** PPy/PSS coated GCEs (filled circles). The sparse data points for Bare GCE is due to reaching the minimum voltage very early in the experiment. Black and blue lines are instantaneous and progressive nucleation models, respectively, as calculated by current-transformed Scharifker-Hills equations.[3]

<u>**Section S3: Gas evolution measurement setups and electrolyte tanks after cycling**</u>

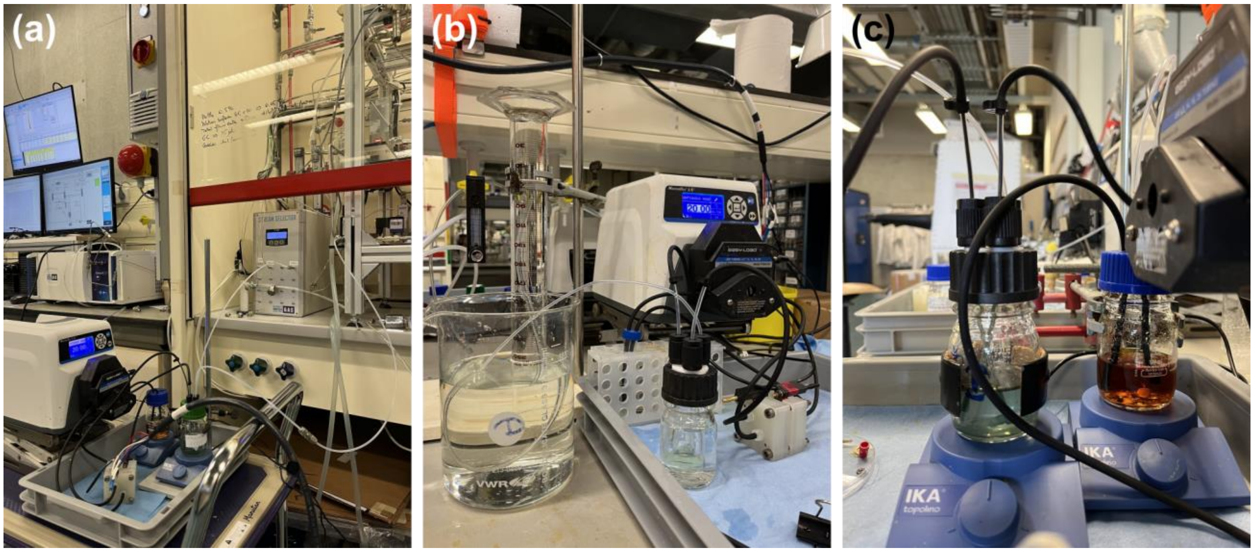

Figure S3. (a) The in-line GC setup used for the cycling tests. **(b)** The inverted graduated cylinder setup where evolved gasses can be collected in the headspace. In both cases the tank is attached to the flow cell with PFA and rubber tubes. **(c)** The electrolyte tanks after cycling, showing the deep orange color of the posolyte and the green precipitates in the negolyte tank and on the walls of the PFA tubes.

Section S4: Calibration curve of the GC for H_2

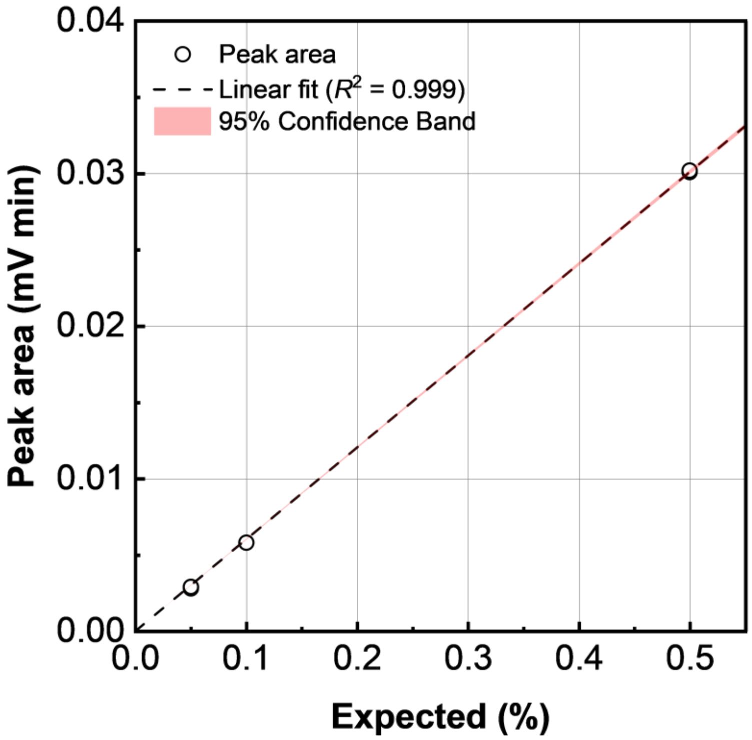

Figure S4. Calibration curve of the GC for H_2. The concentration is set by diluting the calibration gas with N_2. Slope of the linear fit = 0.06021. Measured 5 points per concentration.

__**Section S5: HER experiments on PPy/PSS coated GCEs**__

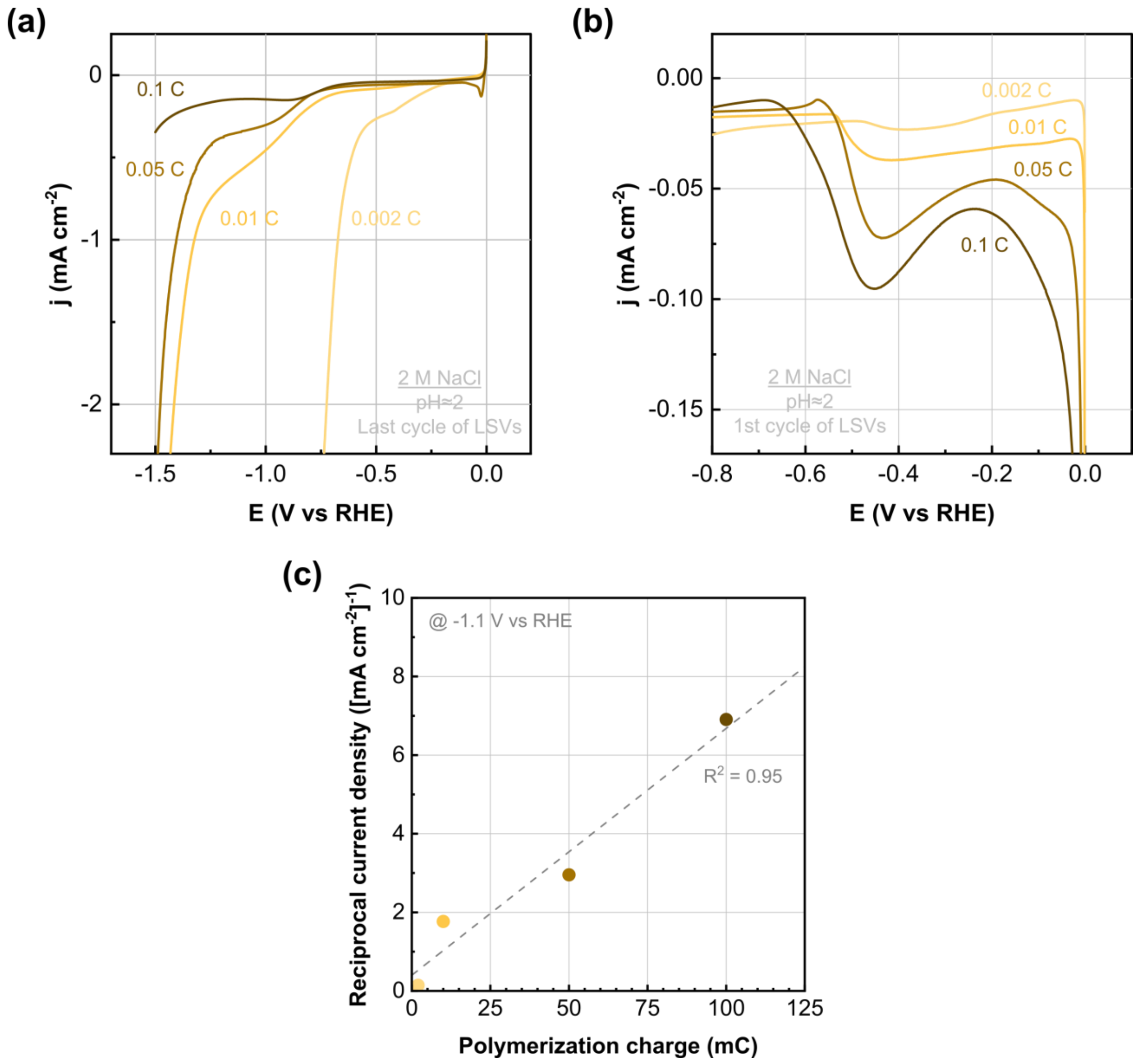

Figure S5. (a) Zoomed in plot of the PPy/PSS coatings to better observe the limiting current region. **(b)** Reduction waves of the coatings as seen in their first LSV cycle. Because the LSVs are conducted at a potential where coatings are in their reduced form, charge is initially spent on polymer reduction. The area under the curve is larger for coatings with higher polymerization charge, indicating a thicker coating. **(c)** Reciprocal of the HER current (in absolute values) plotted against the polymerization charge.

Section S6: Plating/stripping dynamics under optical microscope

For the microscopy experiment, the electrode is a glassy carbon plate (1 cm^2) that is successively polished with 0.3 μm and 0.05 μm alumina slurries on a polishing pad. We use a micro-electrochemical cell under an optical microscope, with experimental conditions similar to the Fe-plating on the RDE electrode (**Figure S2**). The counter electrode is an iron foil ring surrounding the active area, and an RHE reference electrode is placed outside the counter electrode area. The working electrode active area is limited to 0.196 cm^2 (5 mm diameter – same as the RDE) by masking with Kapton tape. The solution is 0.5 M $FeCl_2$ in 2 M NaCl (pH≈2). For charge and discharge, we set the current density at 5 mA cm^{-2}, limit the charging step to 9 mins and discharge until 0.2 V potential cutoff is reached. This protocol is performed two times.

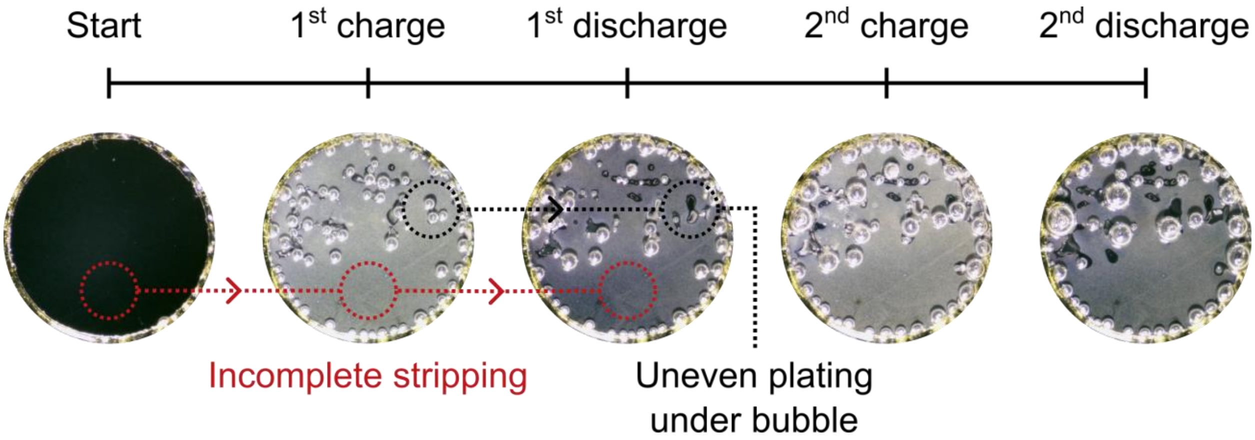

Figure S6. Iron plating/stripping imaging on a bare glassy carbon plate electrode with snapshots at the end of each charge/discharge step.

Section S7: Hydrogen production rate of the cells

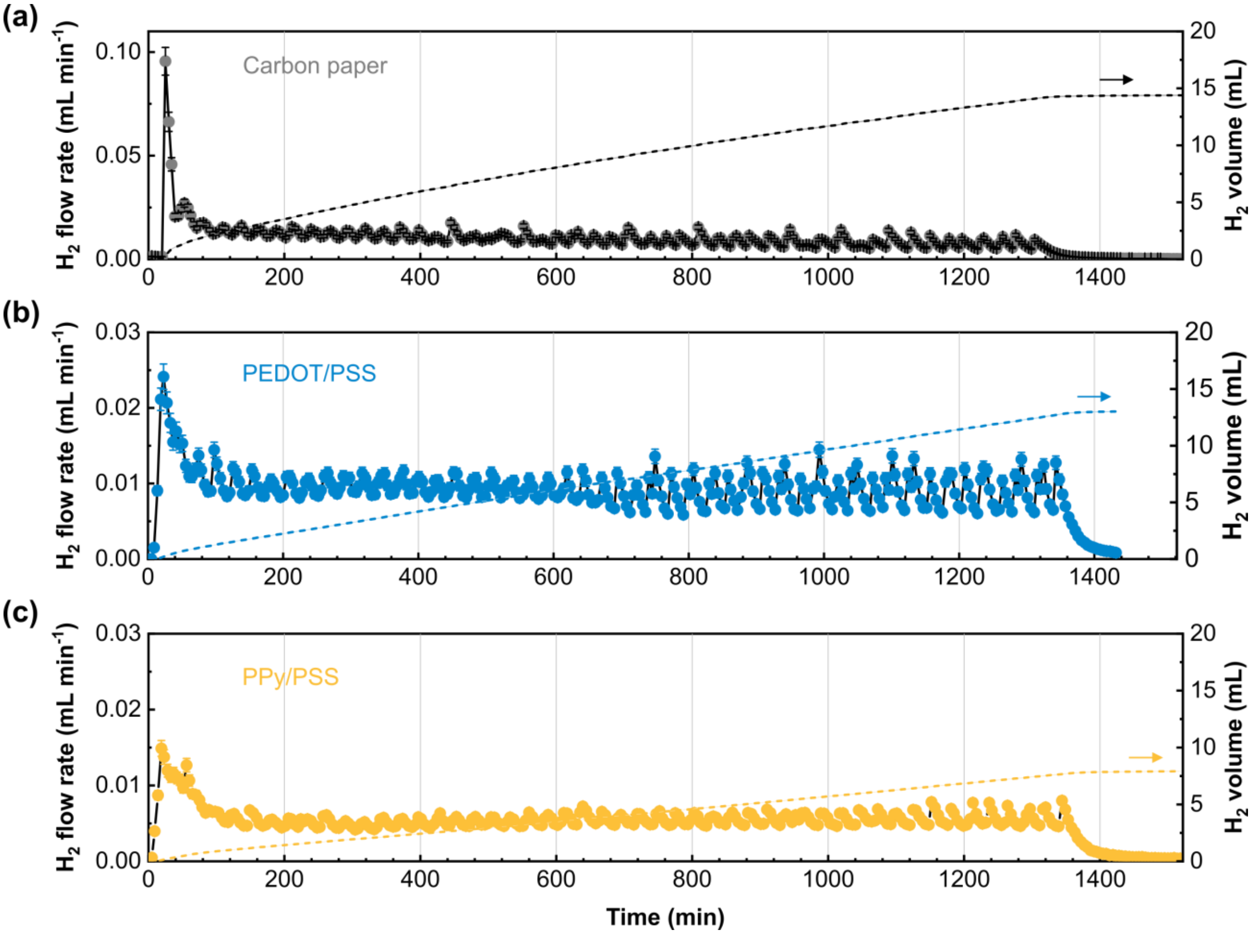

Figure S7. Flow rate and total evolved volume of hydrogen for the **(a)** Uncoated, **(b)** PEDOT/PSS coated, and **(c)** PPy/PSS coated carbon paper electrodes as determined by the in-line GC setup during battery cycling. The scatter plots are measured points, full lines are to guide the eye between the points. Error bars are the error of the flow meter (7%) at the flow range employed in the experiments. Dashed lines are the integral curve of the measured points and give the total evolved H_2 volume (right axes) until that time instance. The shaded areas around the dashed lines are the propagated quadratic error of the flow meter with the integral operation.

Section S8: Long term cycling experiments

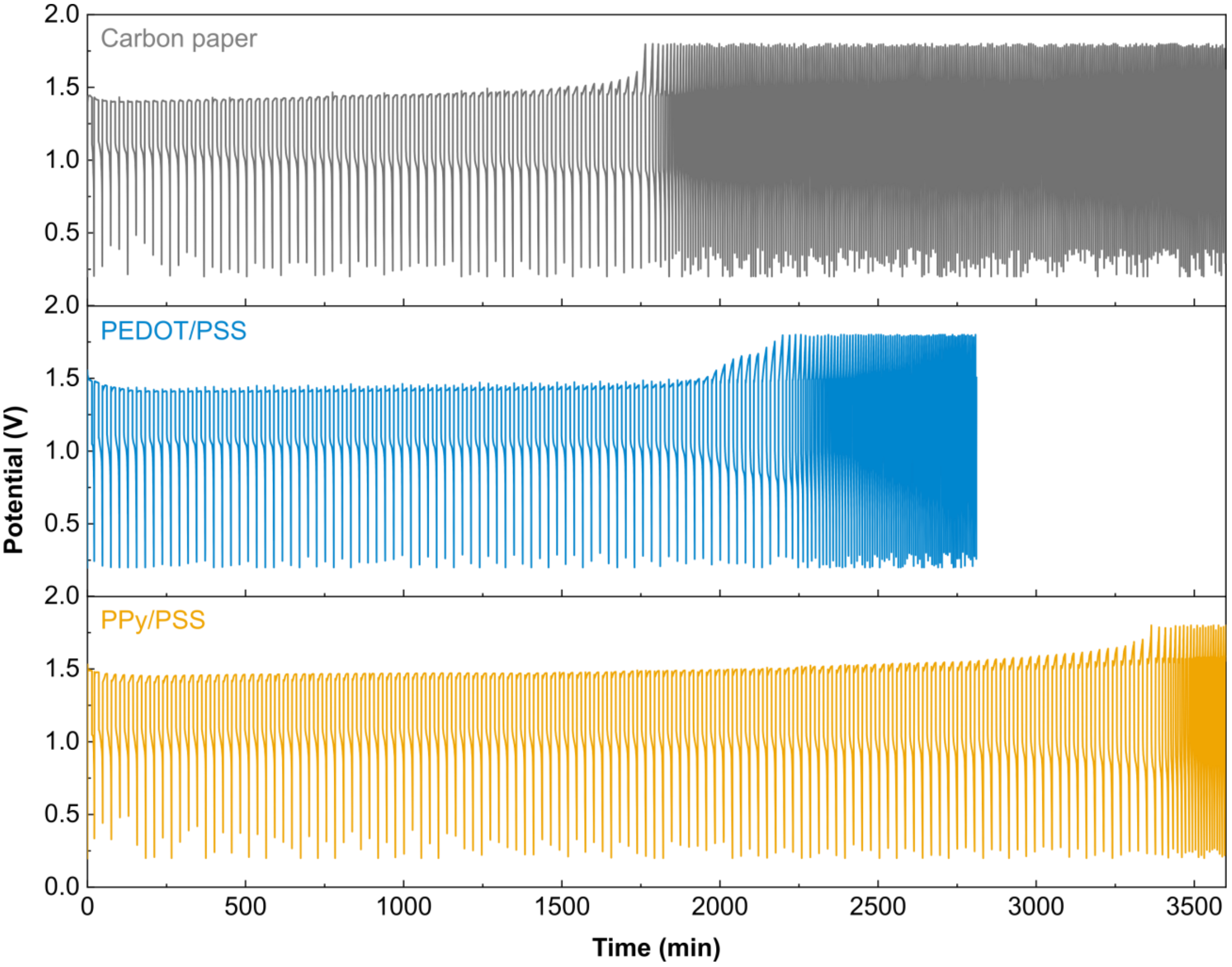

Figure S8. Voltage-time curves of the long term cycling experiments at 25 mA cm^{-2} with 15 mAh charge limit.

Table S1. Efficiencies of the cells with uncoated, PEDOT/PSS and PPy/PSS coated carbon paper electrodes for the long term cycling experiments. CE, VE and EE are averaged over the cycle number until failure ($n_{Failure}$). The errors are one standard deviation from the mean.

**Sample**	$n_{Failure}$	**CE (%)**	**VE (%)**	**EE (%)**
Carbon paper	66	90.82 ± 5.62	63.72 ± 5.01	57.82 ± 5.34
PEDOT/PSS	82	90.92 ± 4.87	66.71 ± 5.40	60.77 ± 6.75
PPy/PSS	125	92.85 ± 3.48	61.39 ± 5.14	56.98 ± 5.11

Section S9: Cross section SEM micrographs of an electrode after cycling

The iron residues in the negative electrode immediately oxidize when the cells are disassembled under ambient atmosphere. We observed that if water can be removed from the structure without oxygen contact, then the oxidation can be greatly reduced. To prepare the sample for imaging, the cell was pumped with water after cycling tests to get the electrolyte out of the pores and immediately transferred to the antechamber of the glovebox. Here the cell dried for overnight under vacuum and then was transferred into the main chamber of the glovebox. The cell was disassembled under N_2 atmosphere and the negative electrode was washed with N_2 purged isopropyl alcohol to remove weakly bound particles. After drying under vacuum, the electrode has alternating green lines that is reminiscent of the flow field channels and ferrous hydroxide precipitation. The dried electrode was cut with an ion-miller (IM4000II, Hitachi) with an accelerating voltage of 6 kV, discharge voltage of 1.5 kV, and under 0.1 sscm N_2 flow. The electrode was milled for 2 hours. The ion-milled area was imaged with a field emission scanning electrochemical microscope (Phenom Pharos G2, ThermoScientific) at 10 kV accelerating voltage.

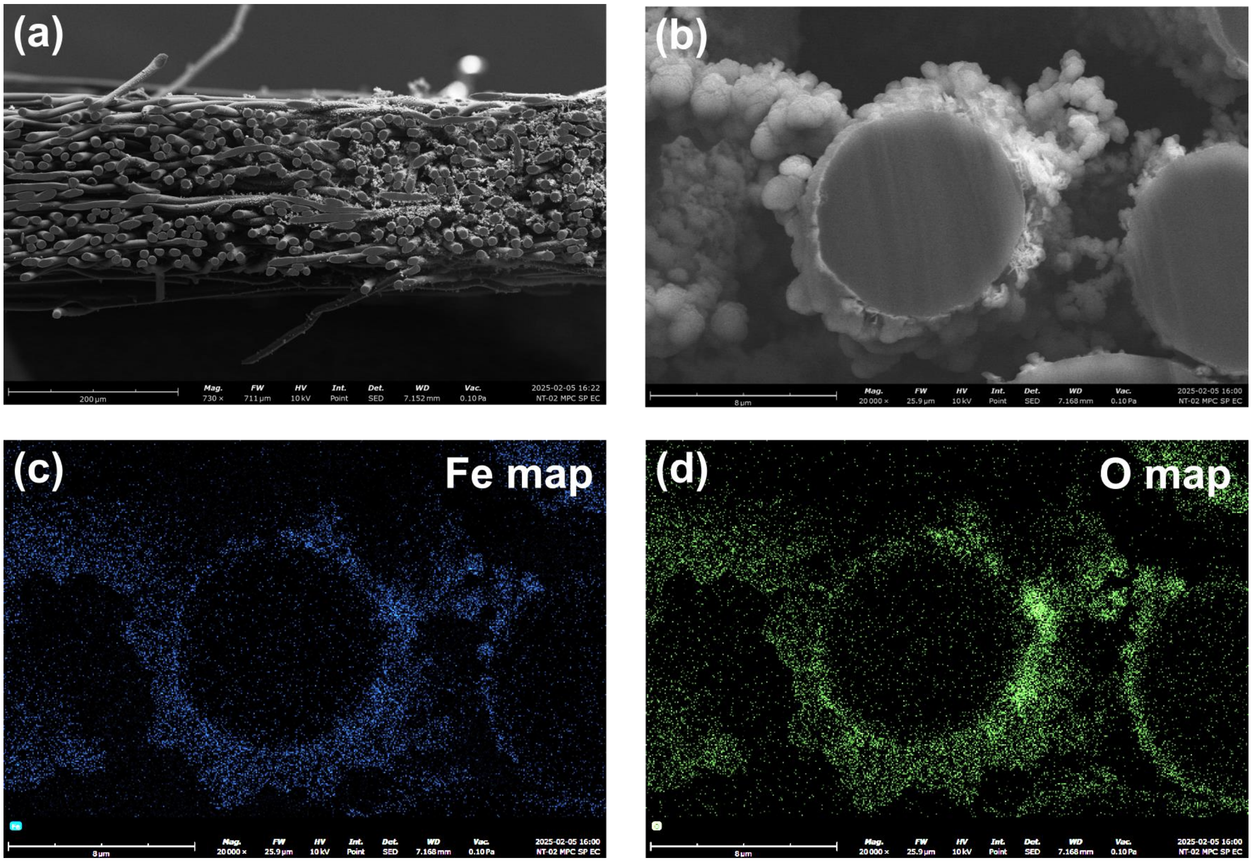

Figure S9. Cross section micrographs of a PPy/PSS coated carbon paper after 50 cycles. **(a)** 730X magnification of the electrode, showing two distinct regions. The right side of the electrode is clogged with residues. **(b)** Zoomed in image of a fiber from the clogged region at 20KX magnification. The PPy/PSS coating cannot be resolved at this magnification. **(c)** Iron and **(d)** Oxygen EDX maps of the same region as in **(b)**. The residues consist of iron and its oxides. The brightness of the EDX maps are adjusted to improve the visibility.

References

[1] I. Gimenez-Garcia, A. Forner-Cuenca, *Electrochimica Acta* **2024**, *498*, 144509.
[2] T. Rapecki, Z. Stojek, M. Donten, *Electrochimica Acta* **2013**, *106*, 264.
[3] Y. Yuan, G. Luo, N. Li, *RSC Advances* **2021**, *11*, 31526.
